# HCMV infection of terminally differentiated neurons disrupts microtubule organization, resulting in neurite retraction

**DOI:** 10.1128/spectrum.01198-25

**Published:** 2025-10-08

**Authors:** Jacob W. Adelman, Andrew T. Sukowaty, Kaitlyn J. Partridge, Jessica E. Gawrys, Allison Akins, Scott S. Terhune, Allison D. Ebert

**Affiliations:** 1 Department of Cell Biology, Neurobiology, and Anatomy, Medical College of Wisconsin 5506, Milwaukee, Wisconsin, USA; 2 Department of Microbiology and Immunology, Medical College of Wisconsin 5506, Milwaukee, Wisconsin, USA; 3 Marquette University and Medical College of Wisconsin Department of Biomedical Engineering, Medical College of Wisconsin 5506, Milwaukee, Wisconsin, USA; Oklahoma State University College of Veterinary Medicine, Stillwater, Oklahoma, USA

**Keywords:** HCMV, induced pluripotent stem cells, tubulin, paclitaxel, colchicine

## Abstract

Human cytomegalovirus (HCMV) is a prolific human herpesvirus that infects most individuals by adulthood. While typically asymptomatic in adults, congenital infection can induce serious neurological symptoms, including hearing loss, visual deficits, cognitive impairment, and microcephaly in 10%–15% of cases. HCMV has been shown to infect most neural cells, with our group recently demonstrating this capacity in stem cell-derived forebrain neurons. Infection of neurons induces deleterious effects on calcium dynamics and electrophysiological function paired with gross restructuring of neuronal morphology. Here, we utilize an induced pluripotent stem cell-derived model of the human forebrain to demonstrate how HCMV infection induces syncytia, drives neurite retraction, and remodels microtubule networks to promote viral production and release. We establish that HCMV downregulates microtubule-associated proteins while largely sparing other cytoskeletal elements. Furthermore, we pharmacologically modulate microtubule dynamics using paclitaxel (stabilize) and colchicine (destabilize) to examine the effects on neurite structure, syncytial morphology, and viral release. With paclitaxel, we found improvement of neurite outgrowth, but neither paclitaxel nor colchicine impacted viral titers. Together, these data suggest that HCMV infection-induced disruption of microtubules in human cortical neurons can be partially mitigated with microtubule stabilization, suggesting a potential avenue for future neuroprotective strategies.

**IMPORTANCE:**

Infection by human cytomegalovirus (HCMV) continues to cause significant damage to human health. In the absence of a vaccine, vertical transmission from mother to fetus can result in profound neurological damage impacting quality of life. These studies focus on understanding the impact of HCMV infection on forebrain cortical neurons derived from induced pluripotent stem cells (iPSCs). We show that infection results in loss of neurite extension accompanied by cell-to-cell fusion. These pathogenic changes involve HCMV infection-mediated disruption of the microtubule network in iPSCs from different patient backgrounds. The microtubule stabilization agent paclitaxel partially protected neurite length and altered syncytia morphology without impacting viral replication. This work is part of our continued efforts to define putative strategies to limit HCMV-induced neurological damage.

## INTRODUCTION

Human cytomegalovirus (HCMV) is a pervasive pathogen that is estimated to infect between 40% and 90% of adults worldwide (1, 2). As a human beta-herpesvirus, HCMV infection is lifelong, occurring in waves of dormancy (latency) and reactivation. Furthermore, transfer of infection can occur either through post-natal exposure to infected bodily fluids (horizontal transfer) or vertically from parent to fetus *in utero*. HCMV infects a wide range of cell types, including fibroblasts, various subtypes of endothelial and epithelial cells, placental trophoblasts, and hematopoietic precursors (3–7). Neural cells have also been established as a site of infection. Neural progenitor cells (NPCs) are central nervous system-specific stem cells and a key site of infection within the human brain (8–10). Other cell types demonstrated to sustain infection include astrocytes (11), microglia (12), oligodendrocytes (13), and ependymal cells (14). The potential for neurons to become infected has, however, been debated within the field (11, 15–17). Recently, both our group and others have demonstrated that terminally differentiated neurons are susceptible to infection regardless of whether their progenitors were induced pluripotent stem cell (iPSC) or fetal stem cells (18–21). Furthermore, infection has been demonstrated to have functional impacts on neurons, including alterations to calcium signaling and reduced action potential generation (18, 21). While these studies have been helpful in characterizing HCMV-induced functional changes in neurons, further evaluation is needed to understand how these cells change structurally.

The neuronal cytoskeleton is composed of three primary elements: actin microfilaments, neurofilaments, and microtubules. Together, these structures function to resist structural deformation, maintain intracellular organization, and allow the cell to interact with its surrounding environment. Actin microfilaments are composed of polar, filamentous actin (F-actin) strands that offer a quick means for neurite expansion and retraction in neurons. Typically, actin structures are found near the cell membrane and enable dynamic interaction with the surrounding environment via filopodia and lamellipodia formation (22–24). Synaptic structures are also organized specifically by actin microfilaments (23). Finally, actin filaments are also capable of inducing cell movement via the associated motor protein myosin. Neurofilaments are the neuron-specific members of the intermediate filament protein family. Compared with actin microfilaments and microtubules, neurofilaments are not polar and therefore have no associated motor proteins. Neurofilaments primarily serve to maintain cell structure and, secondarily, facilitate neuronal function. At axonal projections, neurofilaments arrange to increase radial diameter, improving the conductivity of electrical depolarizations (action potentials) (25–28). Finally, microtubules compose the third branch of the neuronal cytoskeleton and perhaps serve the most varied roles in neuronal physiology.

Microtubules are hollow, polar, cylindrical structures that are composed of tubulin proteins. Tubulins are a protein superfamily with three primary variants: alpha, beta, and gamma. α- and β-tubulins form heterodimers that are the primary components of microtubules with a structure arising from the repeating, organized assembly of the dimerized proteins (29). αβ dimers bind guanosine diphosphate or triphosphate functional groups that dictate microtubule assembly (30, 31). Microtubules are further strengthened through the binding of microtubule-associated proteins (MAPs) such as Tau and MAP2. MAPs bind microtubules longitudinally, acting both to crosslink individual heterodimers and connect cytoskeletal elements (32, 33). At the negative end of the microtubules, αβ dimers are stabilized using a combination of γ-tubulin and negative-end binding proteins (34). γ-tubulin also plays a key role in nucleation for microtubules at the centrosome (or, to a lesser degree, at the Golgi Apparatus), coordinating with other gamma-tubulin complex components (35, 36). Microtubule nucleation events occur at a microtubule-organizing center. These structures act as a hub for microtubule extension throughout the whole cell and serve a key role in coordinating microtubule function.

Microtubules form an intracellular “highway” system, facilitating the movement of cellular components to their proper destinations. This process is mediated by microtubule-associated motor proteins such as dynein and kinesin to move cargo. This cargo can wildly range in size, from objects as small as viral particles to those as large as entire organelles (37). In neurons, microtubules are indispensable for the trafficking of neurotransmitter-containing synaptic vesicles (38) and for facilitating energy distribution via the trafficking of mitochondria (39). Additionally, microtubules are key to maintaining neuronal morphology, whether semi-statically at the soma and axon or dynamically within dendrites (40). Outside of these roles, microtubules also have a putative role in intracellular signaling cascades (41).

Here, we examined how HCMV infection alters the neuronal cytoskeleton in the context of iPSC-derived forebrain neurons from apparently healthy individuals. We observed significant structural alterations in neuronal morphology occurring between 2 and 14 days post-infection (dpi) with a significant impact on MAP expression. Low-dose pharmacological microtubule modulation impacted neurite length, but it had no impact on viral titers. Taken together, these data demonstrate that a low concentration of microtubule modulation does not alter HCMV infection in iPSC-derived neurons but does help neurons maintain their structure, suggesting that microtubules may be valuable targets for mitigating the neuronal effects of HCMV infection.

## RESULTS

### HCMV infection alters neurite length, localization of tubulin, and expression of microtubule-associated genes

**Fig 1:**
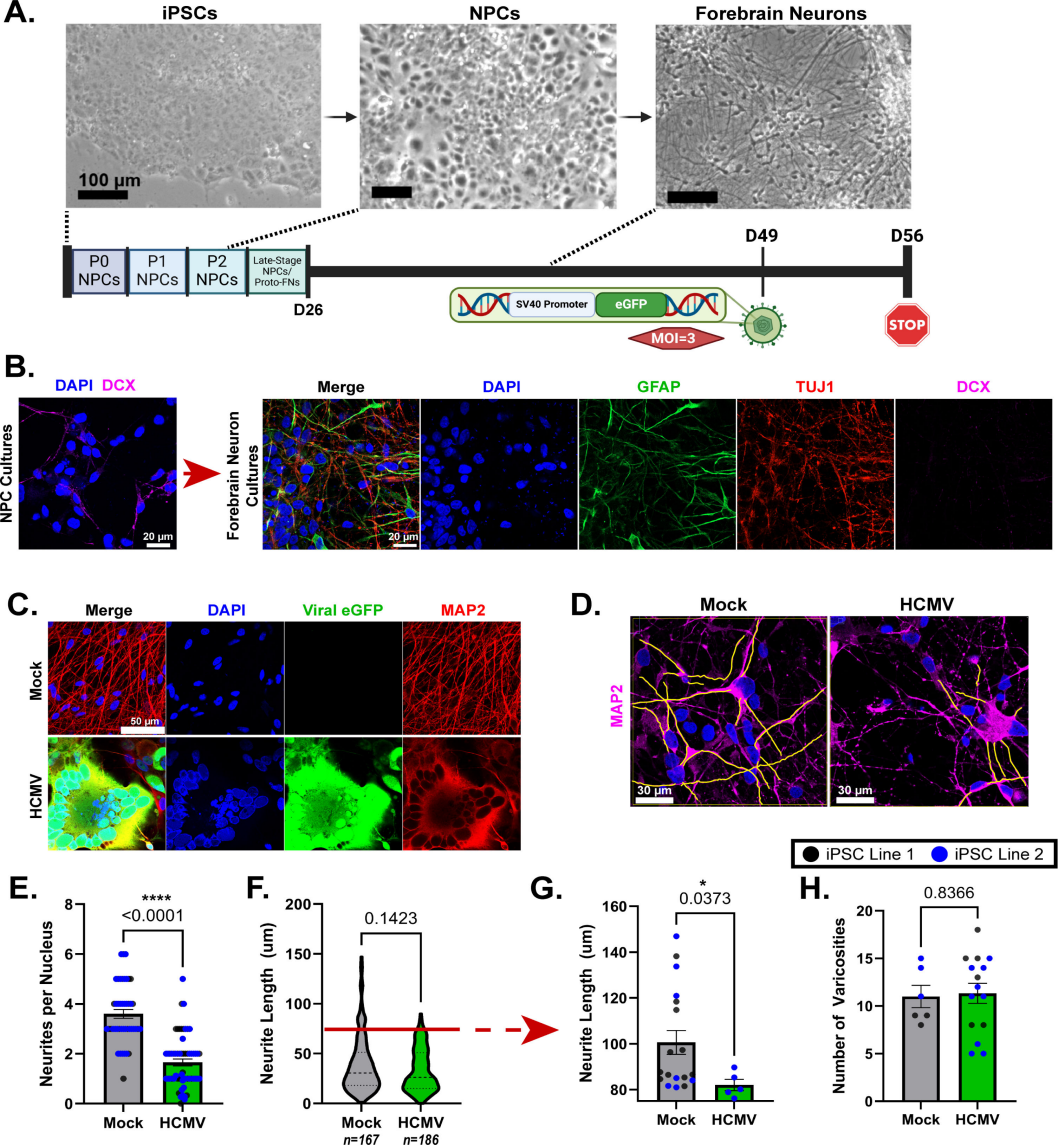
iPSC-derived forebrain neurons infected with HCMV demonstrate altered neurite morphology. (**A**) Schematic demonstrating derivation of forebrain neurons from iPSCs (scale bars = 100 µm). HCMV clinical variant TB40/E-eGFP encodes an eGFP fluorescent indicator protein controlled by an SV40 promoter. Neurons infected in these experiments were done at an MOI of 3 IU/cell. (**B**) Representative images of NPC cultures labeled with immature neuron marker DCX and forebrain neuron cultures stained for GFAP (astrocyte marker), TUJ1 (neuronal marker), and DCX. (**C**) Mock-treated and HCMV-infected forebrains demonstrating typical infection-induced syncytial morphology. (**D**) Representative images of manual neurite traces (yellow) using MAP2 staining in mock- and HCMV-treated forebrain neurons. (**E**) Evaluation of neurites in both mock and infected neurons, expressed as neurites per nucleus (dots represent individual cells/syncytia). (**F and G**) Neurite length measures derived from the paradigm shown in panel **D **(dots represent individual neurites). Processes measuring ≥75 µm (above red bar in F) were isolated and plotted to determine the effects of HCMV infection on longer neurites. (**H**) The frequency of varicosities in neuronal projections was plotted comparing mock to HCMV-infected samples. All data are presented as mean ± SEM. Normalcy of data distribution was determined using D’Agostino-Pearson, Anderson-Darling, Shapiro-Wilk, and Kolmogorov-Smirnov testing. An unpaired *t*-test was conducted to evaluate statistical significance in panel H, as the distribution was normal. Due to atypical distribution, Mann-Whitney tests (nonparametric) were used to analyze statistical differences in panels E–G. *P < 0.05, **P < 0.01, and ***P < 0.001.

We used four independent and unrelated iPSC lines with a minimum of three differentiations for each line to assess the impact of HCMV infection on human neuron structure. To generate forebrain neurons, we differentiated iPSCs into an NPC stage and subsequently patterned NPCs toward a forebrain neuron lineage (Fig. 1A). Consistent with our previous work (18), neurons were infected with bacterial artificial chromosome (BAC)-derived HCMV TB40/E-eGFP (42, 43) at a multiplicity of infection (MOI) of 3 infectious units per cell at day 51 of differentiation (Fig. 1A). To validate the maturity of our culture system, we evaluated expression of the early neuronal marker doublecortin (DCX; Fig. 1B). Further differentiation demonstrates that DCX expression is lost as the neurons mature to express neuron-specific β III tubulin (TUJ1) and exhibit the expected morphology with long thin processes (Fig. 1B). As we have shown previously (18), cultures also contain an astrocyte population (glial fibrillary acidic protein [GFAP], Fig. 1B). Together, these data demonstrate consistent differentiation of mature neural populations. Finally, using a genetically encoded eGFP reporter HCMV, we determined sites indicative of infection at 7 dpi (Fig. 1C).

Initial observation of infected cultures revealed significant alteration to neuronal structure, including formation of syncytia and a visual change to neurites (Fig. 1C; Video S1). Using neurite tracing analysis in combination with MAP2 staining, we found that neurite quantity per nucleus was significantly decreased when neurons were infected with HCMV compared to mock conditions (Fig. 1D and E). Neurite tracing also revealed a trend toward decreased neurite length in infected cultures (Fig. 1D and F). Furthermore, when longer neurites (those equal to or greater than 75 µm; bottom threshold indicated by the red line in Fig. 1F) were isolated from the total population, infection significantly decreased neurite length (Fig. 1G). Additionally, lengthy neurites occurred more frequently in mock-treated cells (*n* = 18) relative to their infected counterparts (*n* = 5; Fig. 1G). Notably, neurites ≥75 µm were only present in one of the two lines post-infection (Fig. 1G). To confirm that the decrease in neurite length was not due to overt axonal degeneration, we measured axonal varicosities (e.g., beads on a string [44]), a phenotype commonly associated with axonal breakdown and degeneration, in observable neurites in mock and HCMV conditions. We did not observe a significant difference, suggesting that HCMV infection is not causing overt neurodegeneration (Fig. 1H). These data are consistent with our previous findings showing no significant increase in cell death in HCMV-infected cortical neuron cultures (18).

**Fig 2:**
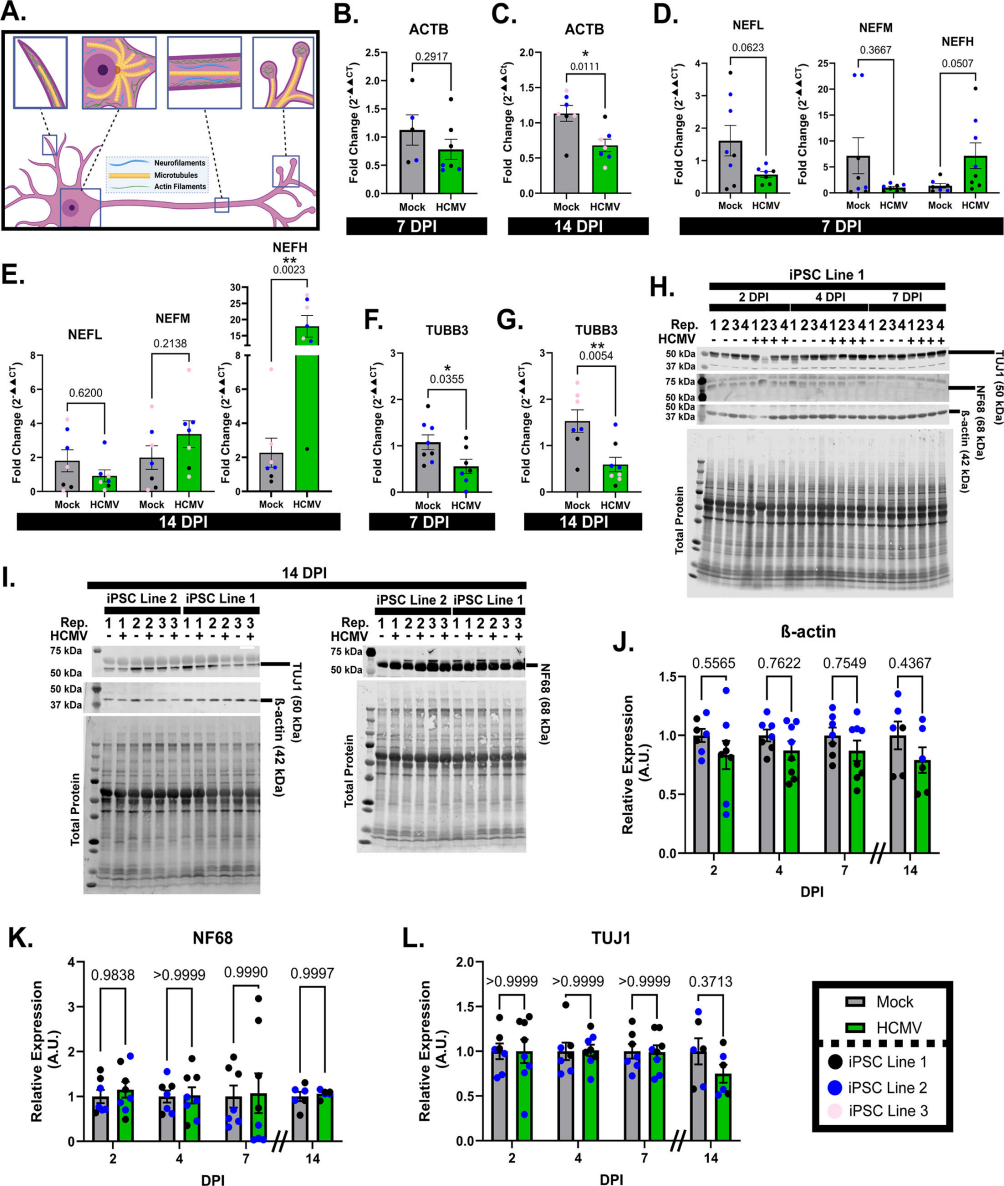
HCMV effects on cytoskeletal transcripts and proteins. (**A**) Schematic showing the various components of the neuronal cytoskeleton. (**B and C**) HCMV infection induces significant downregulation of actin mRNA at 14-dpi, but not at 7 dpi. (**D and E**) Infection of forebrain neurons does not consistently alter neurofilament transcripts at either 7 or 14 dpi. (**F and G**) TUBB3 (neuron-specific βIII tubulin) gene expression is significantly downregulated upon infection at 7 and 14 dpi. (**H–L**) Western blot images and quantification demonstrating that infection does not induce significant changes in actin, neurofilament 68 (NEFL), or tubulin (TUJ1) protein expression at 2, 4, 7, and 14 dpi. Western blot data are normalized to total protein. All data are presented as mean ± SEM. Panels B, D (NEFL and NEFH), E (NEFM), F, and G were analyzed using unpaired *t*-tests. Panels C, D (NEFM), and E (NEFL and NEFH) were analyzed using Mann-Whitney tests due to abnormal distribution. An ordinary two-way analysis of variance (ANOVA with Šídák multiple comparisons test (single pooled variance) was utilized for panels H–J. *P < 0.05, **P < 0.01, and ***P < 0.001.

To further investigate the neurite retraction phenomenon, we sought to evaluate the effects of HCMV on various elements of the neuronal cytoskeleton, including actin filaments, neurofilaments, and microtubules (Fig. 2A). Assessment of gene expression for these structural components in neurons from multiple iPSC lines was first conducted using RT-qPCR. The effects of HCMV on β-actin (ACTB) transcripts showed no significant change at 7 dpi but a significant decrease at 14 dpi (41%; Fig. 2B and C). Neurofilament subunits showed a non-significant HCMV-induced reduction in both light (NEFL) and medium (NEFM) chain mRNAs at 7 dpi and no consistent change at 14 dpi, but there was an HCMV-induced upregulation of heavy chain (NEFH) transcripts at both timepoints (fivefold at 7 dpi; 17-fold at 14 dpi; Fig. 2D and E). Finally, mRNAs for neuron-specific beta-III tubulin (TUBB3) were found to be significantly downregulated at both 7 (49%) and 14 dpi (62%; Fig. 2F and G). These findings indicate downregulation of both ACTB and TUBB3 gene expressions, but upregulation of NEFH in neurons from multiple iPSC lines.

We next examined the temporal changes in cytoskeletal elements at a protein level. We noted a consistent trend toward downregulation across all timepoints for ACTB expression (Fig. 2H through J), which mirrored a similar trend to the mRNA levels. Neurofilament 68 (NF68, NEFL gene product) was selected to represent the effects of HCMV on neurofilament expression. There was no significant effect of HCMV on NF68 protein expression across all tested timepoints (2, 4, 7, and 14 dpi; Fig. 2H, I and K). We were unable to identify a working antibody to NEFH. Finally, TUJ1 was examined to assess the effects of infection on microtubules. No significant change in TUJ1 expression was noted at any timepoint, though a modest decrease in tubulin was present at 14 dpi (Fig. 2H, I and L). As we see HCMV-induced structural deformations of the neuronal cytoskeleton at 7 dpi (Fig. 1C), our findings indicate that HCMV modulation of cytoskeletal elements likely does not involve altered expression levels of the tested structural proteins. Therefore, we next sought to evaluate proteins that modulate cytoskeletal stability.

**Fig 3:**
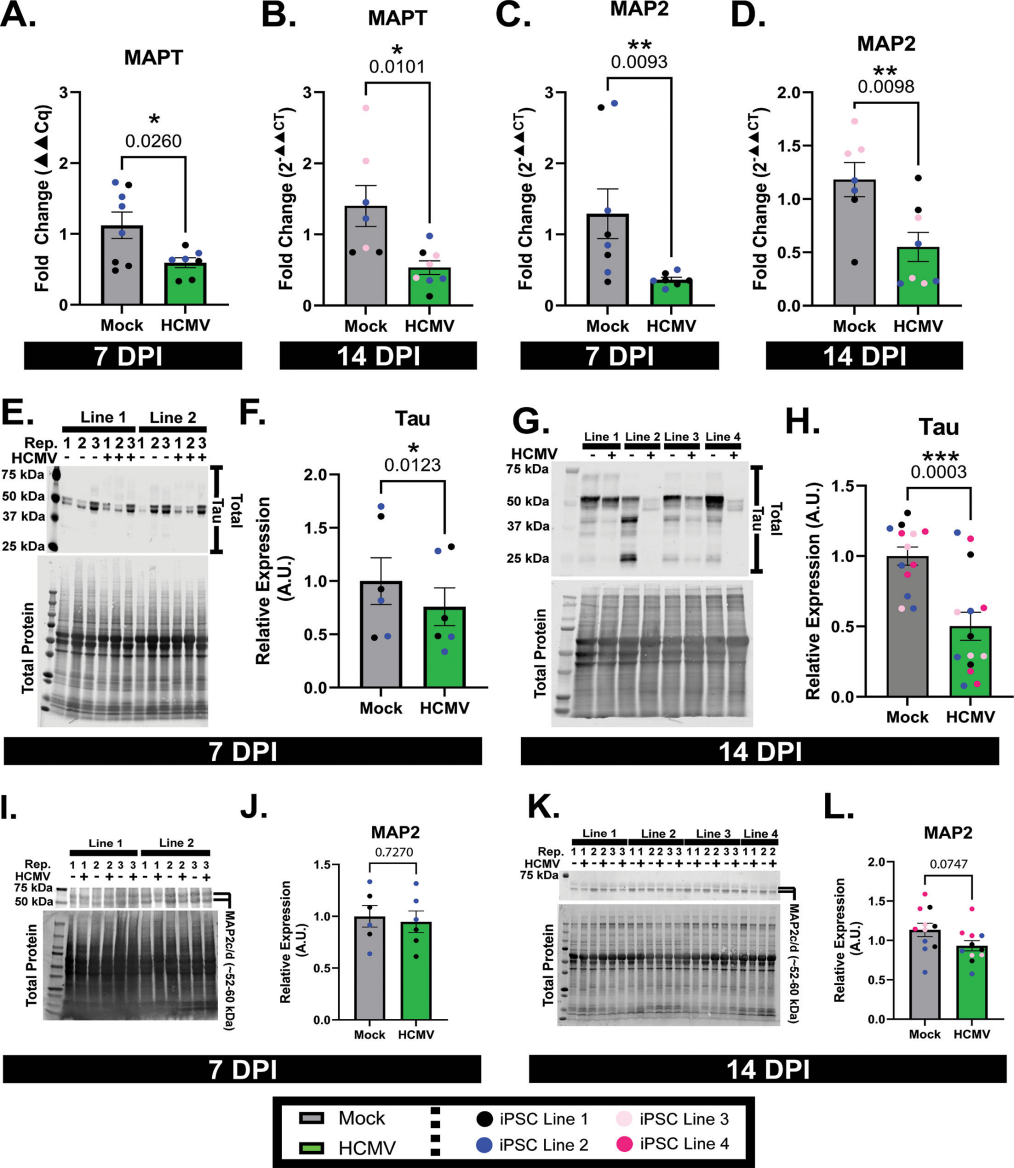
Infection with HCMV alters MAP expression. (**A and B**) MAPT transcripts are downregulated by HCMV infection at both 7 and 14 dpi timepoints. (**C and D**) Infection drives significant decreases in MAP2 mRNA at 7 and 14 dpi. (**E–H**) Western blot quantification and images demonstrated HCMV-dependent decreases in tau (MAPT gene product) expression upon infection at both tested timepoints. (**I–L**) Infection induces a non-significant decrease in MAP2 protein expression at 14 but not 7 dpi. *Note*: The blot in panel K was stripped of antibody and reused, so the total protein stain is repeated from Fig. 2I. Western blot data are normalized to total protein. All data are presented as mean ± SEM. Unpaired *t*-tests were used to analyze statistical differences in panels A, B, and D–J. Due to non-normal distribution, 3C was analyzed using Mann-Whitney testing. *P < 0.05, **P < 0.01, and ***P < 0.001.

Using neurons derived from multiple iPSC lines, we assessed two key MAP genes, MAPT and MAP2, using RT-qPCR. MAPT transcripts were found to be downregulated at both 7 (48% decrease) and 14 dpi (62% decrease; Fig. 3A and B). Likewise, MAP2 transcripts were decreased at both timepoints (72% at 7 dpi and 53% at 14 dpi; Fig. 3C and D). Assessment of total tau protein (MAPT-encoded isoforms) by western blot found significant downregulation at both 7 (24%) and 14 dpi (50%; Fig. 3E through H). MAP2 protein expression was not changed at 7 dpi (Fig. 3I and J) but was modestly decreased at 14 dpi by 18%; Fig. 3K and L). Together, these data show decreased MAP expression levels, suggesting that neuronal microtubule stability may be impacted by HCMV infection.

**Fig 4:**
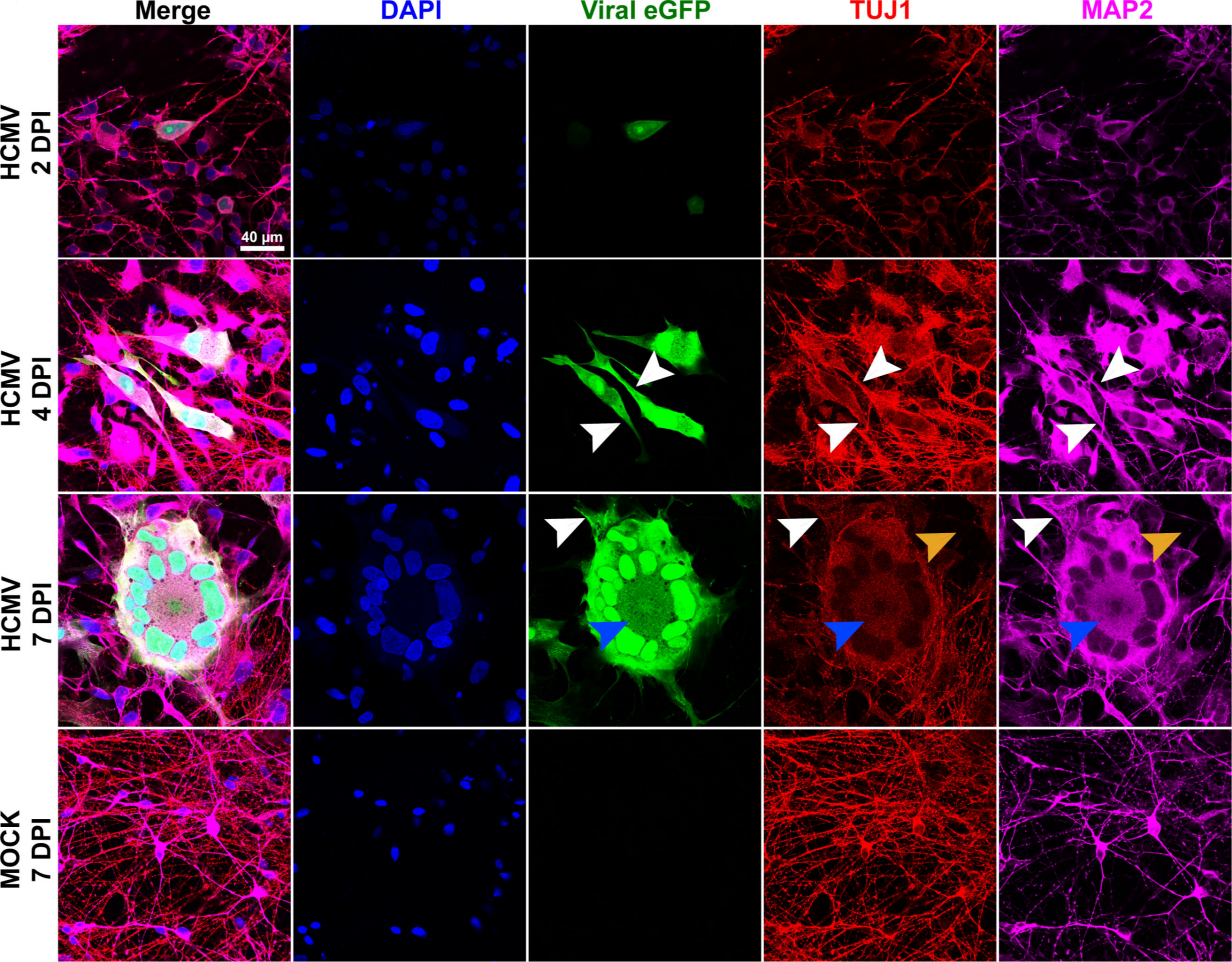
Time course evaluation of infection over the first 7 days. Forebrain neurons infected with HCMV begin to show eGFP-positive plaques as early as 2 dpi. Furthermore, neurite changes can be observed as early as 4 dpi relative to mock cultures. These findings include thickened processes (denoted by white arrows). At 7 dpi, tubulin and MAP2 staining appeared punctate in the syncytia core (blue arrows), rather than filamentous as examined elsewhere. Furthermore, regions lacking neurites were more common in the areas adjacent to the syncytia (orange arrows). Scale bars = 40 µm.

Although there were no significant changes noted in total tubulin protein expression through the first 7 days of infection, neuron structure was altered. We examined neuronal infection at 2, 4, and 7 dpi using immunofluorescence and observed changes in tubulin and MAP distribution within infected cells. First, a subclass of neurites associated with eGFP-labeled cell bodies appeared thickened relative to their mock-treated counterparts (Fig. 4A). This structural change was noticed as early as 4 dpi and continued to be observed within 7 dpi cultures (Fig. 4A, white arrowheads). Furthermore, at 7 dpi, TUJ1 staining within syncytia revealed non-filamentous, punctate signal within the syncytial core (Fig. 4A, blue arrowheads). Finally, we noted areas devoid of neurite processes within infected cultures, especially distal to syncytia (Fig. 4A, orange arrowheads). These data suggest that infection induces a structural phenotype by utilizing the existing tubulin present within a cell. Therefore, we hypothesized that HCMV infection alters microtubule stability rather than affecting overall microtubule abundance.

### Stabilization of microtubules with paclitaxel alters syncytial morphology

We next utilized two compounds known to impact the formation and stability of microtubules to determine the role of microtubule stability in forebrain neuron infection: paclitaxel (Pac), a microtubule-stabilizing agent, and colchicine (Col), a microtubule-destabilizing compound. As both compounds are associated with cell death at high concentrations (45–48), we treated uninfected neurons with varying amounts of Pac and Col for 7 days and evaluated varicosities, neurites per nucleus, and neurite length.

**Fig 5:**
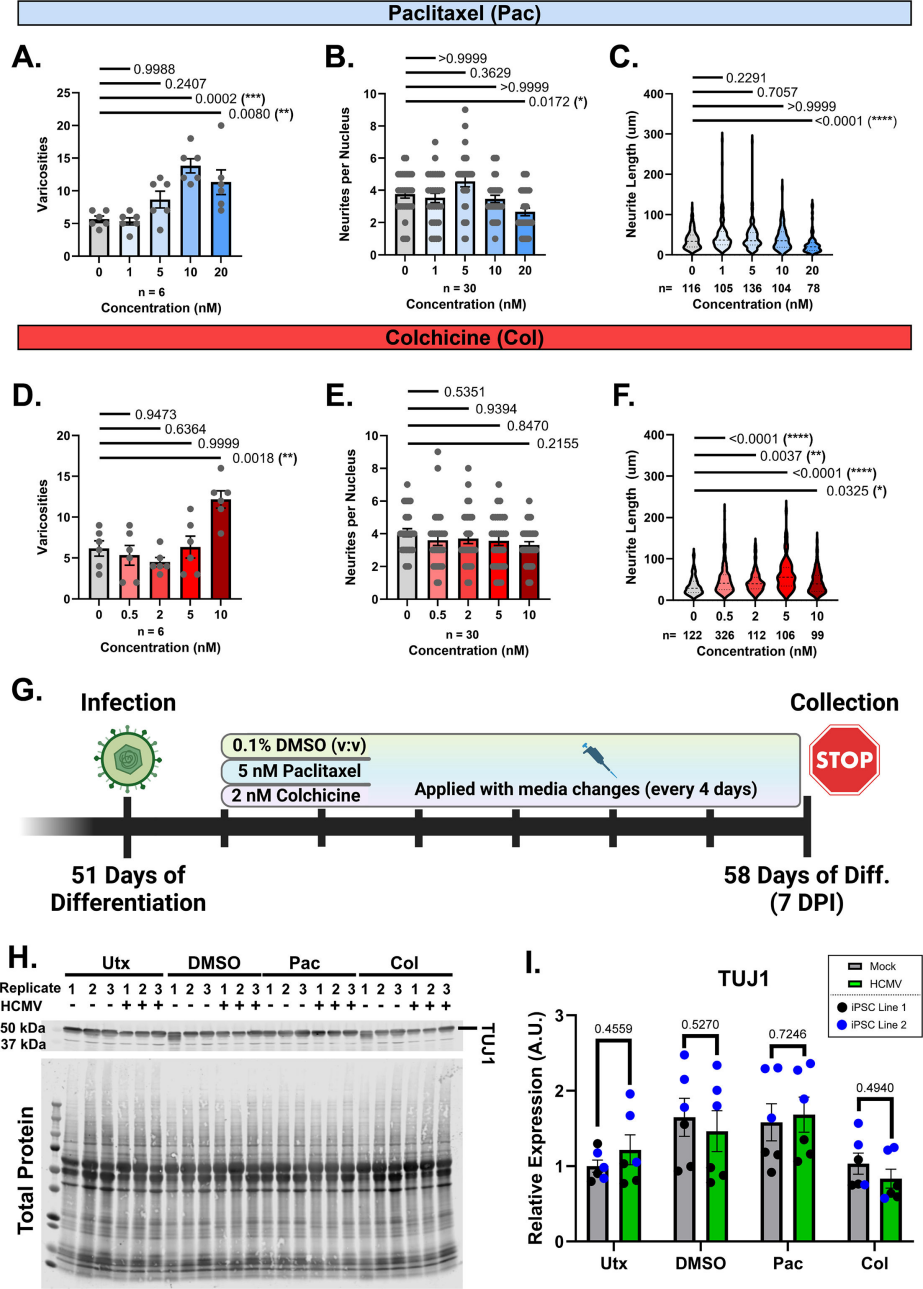
Paclitaxel and colchicine treatment in mock and HCMV-infected forebrain neurons. (**A–F**) Effects of varying concentrations of Paclitaxel (**A–C**) and Colchicine (**D–F**) on mock-treated neuronal cultures, specifically detailing alterations to neuronal varicosities, neurites per nucleus, and neurite length. (**G**) Diagram of the dosing schedule for paclitaxel, colchicine, and dimethyl sulfoxide (DMSO) vehicle for application to forebrain neurons. (**H and I**) Western blot image and analysis for neuron-specific beta III tubulin (TUJ1) show no change in expression with treatment. Western blot data are normalized to total protein. All data are presented as mean ± SEM. All data sets were tested for normalcy before analysis. Ordinary one-way ANOVA with Dunnett’s Multiple Comparison post-hoc tests was utilized for panels A and D due to the normal distribution of data. Panels B, C, E, and F were analyzed using Kruskal-Wallis tests due to non-normal data distributions. An ordinary one-way ANOVA with Tukey’s post-hoc analysis was used to analyze statistical differences in panel I. *P < 0.05, **P < 0.01, and ***P < 0.001.

Treatment with 20 nM Pac significantly increased varicosity formation, neurite loss, and neurite retraction compared to mock (Fig. 5A through C). A total of 10 nM Pac also significantly induced varicosity formation, although it did not significantly impact neurite number or length (Fig. 5A through C). Neither 1 nM nor 5 nM treatment of Pac significantly altered neurite phenotypes compared to mock (Fig. 5A through C). Therefore, we selected 5 nM Pac for subsequent studies. Col treatment revealed few negative effects other than increased varicosities with the 10 nM concentration (Fig. 5D). Col treatment did not have any effect on neurite number at any concentration tested (Fig. 5E) but surprisingly increased neurite length at all concentrations with the greatest increase occurring with 5 nM Col (Fig. 5F). Based on these data, we chose 2 nM Col for the remaining studies.

**Fig 6:**
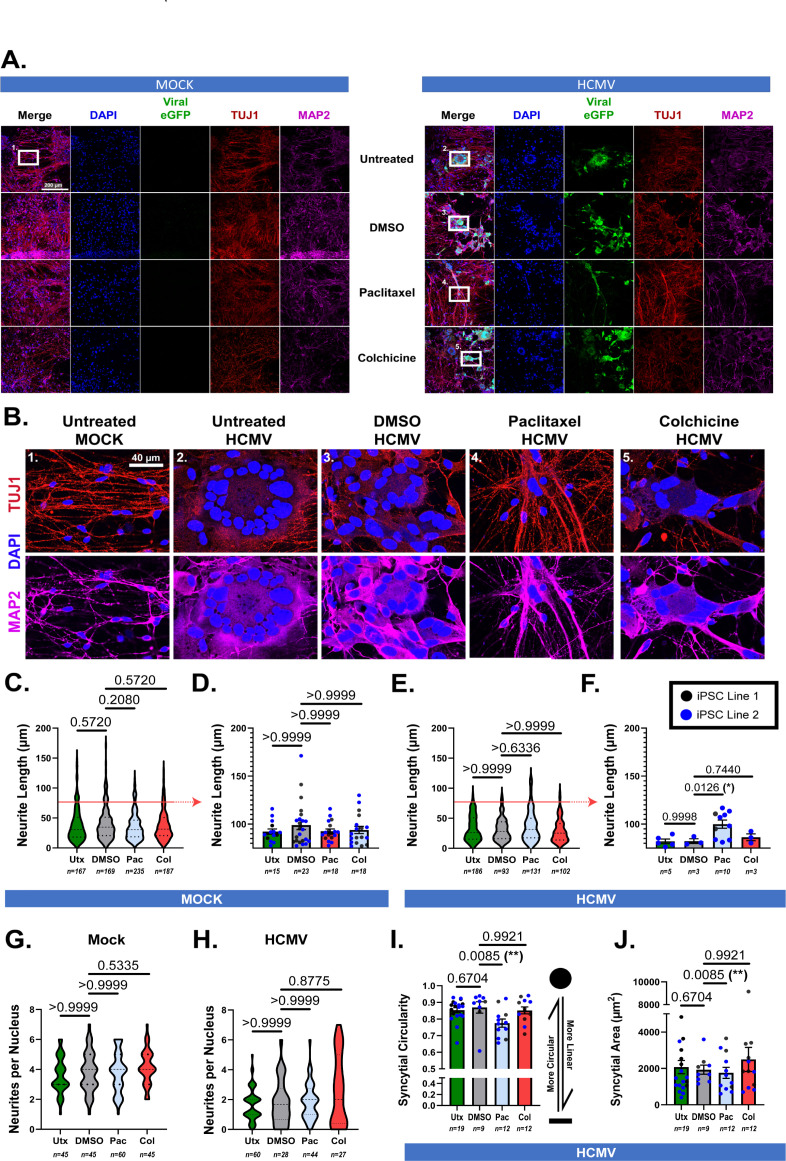
The effects of paclitaxel and colchicine on neurite structure in HCMV-infected forebrain neurons. (**A**) Large-format images (500 µm × 500 µm) detailing the effects of DMSO vehicle, paclitaxel, and colchicine on neurite structure. White boxes are regions of interest (ROIs) that are magnified in panel B. Scale bar = 200 µm. (**B**) Zoomed ROIs from panel A demonstrating tubulin and MAP2-labeled neurites upon treatment with Pac, Col, and DMSO. Scale bar = 40 µm. (**C–F**) Quantification of neurite length (using MAP2 staining) in both mock (**C and D**) and HCMV (**E and F**) conditions (each data point represents a neurite). In D and F, effects of Pac and Col on neurites specifically ≥75 µm (above red bars in C and E) are analyzed relative to DMSO control, as in Fig. 1, and show a significant increase in neurite length in Pac-treated HCMV-infected cells. (**G and H**) Evaluation of the effects of Pac, Col, and DMSO shows no difference in the number of neurites per nucleus in both mock (**G**) and infected (**H**) conditions. (**I**) Syncytial circularity data are reported for HCMV-infected cells treated with DMSO, Pac, and Col, with a scale ranging from 0 (linear) to 1 (circular). Pac-treated HCMV-infected neurons show a significant reduction in circularity compared to the DMSO HCMV-infected condition (each datapoint represents one syncytium). (**J**) Syncytial area is not different across the treatment conditions (each datapoint represents one syncytium). All data are presented as mean ± SEM. Due to abnormal distribution of datapoints, panels C–E and G–J were analyzed using Kruskal-Wallis tests (nonparametric). Data in panel F were analyzed using Brown-Forsythe and Welch ANOVA tests with Dunnett’s T3 multiple comparison corrections. *P < 0.05, **P < 0.01, and ***P < 0.001.

To test the effect of Pac and Col on neuron morphology in the context of HCMV infection, neurons were infected with HCMV and then treated with Pac (5 nM), Col (2 nM), or DMSO (vehicle, 5 nM) for 6 days before collection of cultures at 7 dpi (Fig. 5G). As microtubules are required for HCMV entry and pathogenesis, treatment of the neurons was delayed until 24 hpi. An initial assessment of TUJ1 was conducted at 7 dpi to determine if drug application altered expression. At these treatment concentrations, no significant differences in TUJ1 expression levels were detected between the various treatment groups (Fig. 5H and I). Following this, we investigated tubulin structure via immunofluorescence. Within mock-treated cultures, we detected no significant differences in neurite quantity or length (Fig. 6A and C). We then assessed neurites ≥75 µm in length (above the red line in Fig. 6C) and found no significant differences in neurite length between uninfected untreated (utx) control, DMSO control, Pac, and Col-treated conditions. Likewise, all treatments produced relatively similar amounts of long neurites (utx: *n* = 15, DMSO: *n* = 23, Pac: *n* = 18, and Col: *n* = 18), and there were no significant differences in the number of neurites per nucleus amongst the experimental groups in mock-treated cells (Fig. 6G). For ease of comparisons across treatment groups in Fig. 6C through H, data for the untreated condition are repeated from Fig. 1. Additionally, consistent with Fig. 1F, the DMSO HCMV infection condition showed reduced neurite length compared to the DMSO mock condition (Fig. 6C and E, gray plots). In the HCMV-infected conditions, immunofluorescence demonstrated that syncytia formed in the untreated, DMSO-treated, and Col-treated infected cultures and were similar in shape and size (Fig. 6A, B, I and J), and neurite number and neurite length were not significantly different among these groups (Fig. 6C through H). Interestingly, Pac-treated HCMV-infected neurons formed more linearly organized syncytia than those found within utx, DMSO control, and Col-treated cultures (Fig. 6A, B and I). Furthermore, infected neurons treated with Pac trended toward having longer neurite lengths overall (Fig. 6E), with significantly longer processes ≥75 µm in length compared to HCMV-untreated, HCMV + DMSO and HCMV + Col (Fig. 6F) and with lengths similar to the Pac-treated mock condition (Fig. 6C through F, blue plots). Long neurites were also more abundant among the Pac-treated cells (*n* = 10) relative to utx neurons (*n* = 5), DMSO controls (*n* = 3), and Col-treated neurons (*n* = 3). Interestingly, while longer neurites were still predominantly found in infected cultures derived from iPSC Line 2 (reminiscent of Fig. 1G), we did observe that Pac treatment induced a slight preservation of longer neurites in cells derived from iPSC Line 1, as well (Fig. 6F). Interestingly, Col treatment did not increase neurite length in the presence of HCMV infection as it had done in control conditions (Fig. 6F vs Fig. 5F). Overall neurite counts were not affected (Fig. 6H). These data indicate that microtubule stabilization helps maintain neurite extension and may partially disrupt typical syncytia organization induced during HCMV infection. Considering this, we hypothesized that microtubule modulation could also disrupt viral production.

### Markers of viral production are not significantly altered by modulating microtubule stability

**Fig 7:**
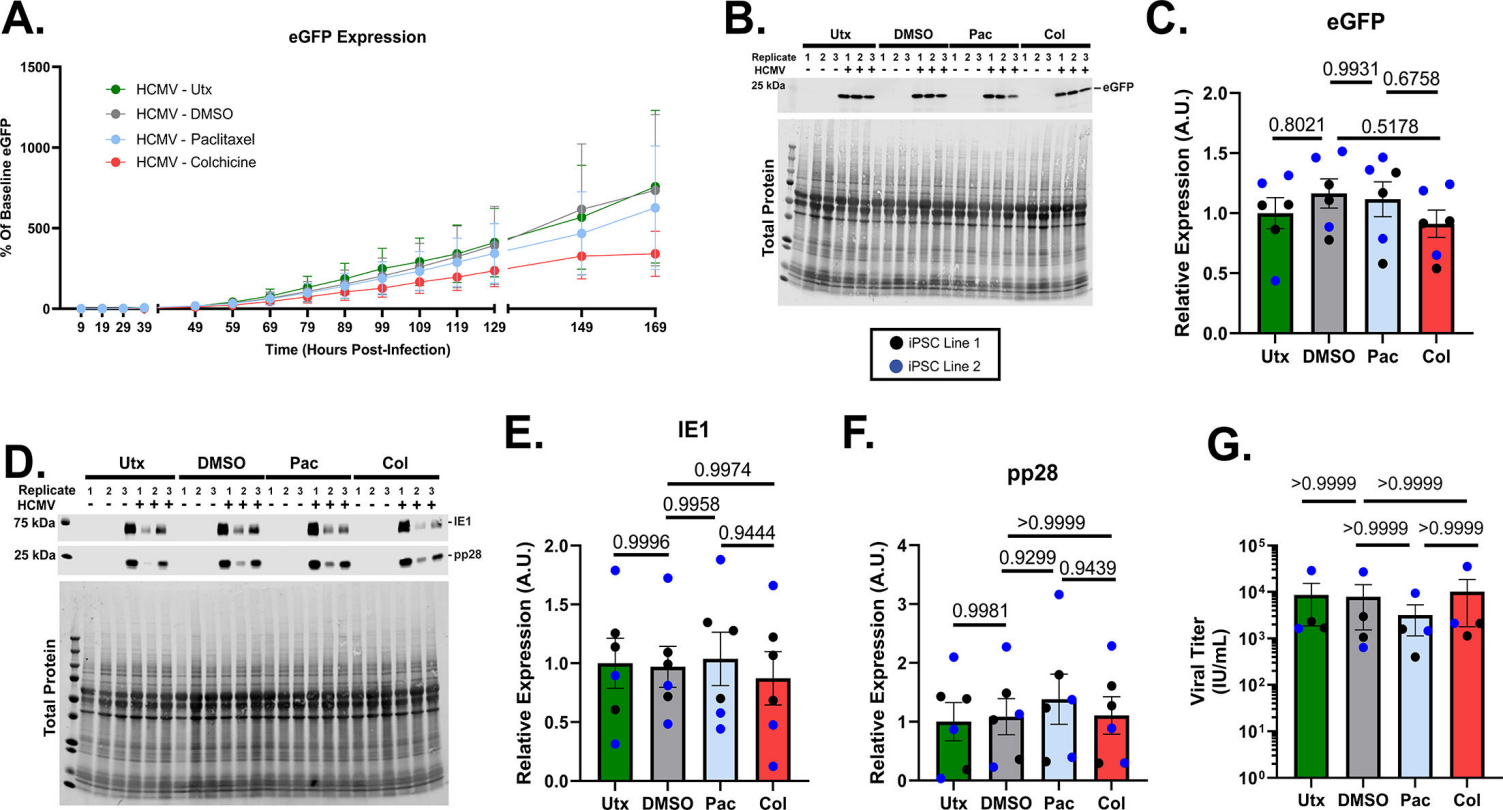
The effects of paclitaxel and colchicine treatments on viral output in HCMV-infected forebrain neurons. (**A**) Viral eGFP expression data were collected over the first 169 hpi for neurons treated with nothing, DMSO, paclitaxel, and colchicine. No significant differences were noted in the amount of eGFP between conditions. (**B and C**) Representative western blot probing for eGFP and associated quantification. Note: This blot was stripped of antibody and reused, so the total protein stain is repeated from Fig. 5B. (**D–F**) Evaluation of viral immediate early 1 protein (IE1) and pp28 with quantification. (**G**) Finally, viral titers were assessed for each condition, revealing no significant changes between DMSO, paclitaxel, and colchicine, though a trend toward reduced viral production was noted in Pac-treated cells. Western blot data are normalized to total protein. All data are presented as mean ± SEM. Panel A was analyzed using a Repeated Measures (RM) with Geisser-Greenhouse correction and Tukey’s Multiple Comparisons post-hoc test. An ordinary one-way ANOVA with Tukey’s post-hoc analysis was used to analyze statistical differences in panels C, E, and F. Due to the non-normal distribution of samples, panel G was analyzed using a combination of Kruskal-Wallis and Dunn’s multiple comparisons tests. *P < 0.05, **P < 0.01, and ***P < 0.001.

Finally, we sought to examine the subsequent impact of microtubule modulation on viral output from infected neurons. Live imaging was conducted for ~7 dpi, with frequent monitoring of eGFP expression within the culture. These traces are depicted in Fig. 7A and did not demonstrate any significant deviations by Pac or Col treatment relative to DMSO-treated and untreated controls. These findings were confirmed upon evaluation of total eGFP expression in 7 dpi cell lysates (Fig. 7B and C). Cell lysates were then probed for relative expression of HCMV immediate early protein 1 (IE1) and late protein pp28, but there were no significant differences between any of the treatment groups for expression of these viral targets (Fig. 7D through F). Finally, viral titers were analyzed using the conditioned medium (CM) from HCMV-infected neurons. Despite changes in syncytia morphology of Pac-treated cells (Fig. 6), there were no significant differences in viral titers between treatment groups (Fig. 7G). Taken together, these data suggest that HCMV infection-induced structural changes can be partially abated by microtubule-modulating treatments without a corresponding impact on viral production.

## DISCUSSION

Microtubules have been known to be associated with HCMV infection for decades. Their relevance in virion entry, capsid trafficking, viral assembly, and, ultimately, egress processes has been thoroughly documented in various cell models (49–54). However, the recent groundswell of interest regarding the effects of HCMV infection in nervous tissue (19, 21, 55–61) requires a reassessment of its impact on microtubules in the context of neurites (axons and dendrites). Due to substantially different structures and functions, the application of findings from fibroblasts onto neurons and NPCs is likely to be problematic. Fortunately, since the discovery of iPSCs, access to human-derived neural cells has increased exponentially, allowing for easier evaluation of HCMV within neurons. Here, we sought to better describe neuron-specific structural phenomena relating to HCMV infection. Specifically, we analyzed effects on the expression of neuronal cytoskeletal components and demonstrated that stabilization of microtubules can mitigate some of the morphological aberrancies induced by infection.

HCMV infection induced significant alterations to neuronal morphology within the first 7 days of infection (Fig. 1C and 4A). Neurites have been described by both our group and others as a focal site of these changes, with projections becoming dissociated from their underlying matrix (21) and ultimately retracting into the neuronal soma (18, 21) (Fig. 1D through G). The other significant morphological change that occurs in HCMV-infected neurons is the formation of multinucleated syncytia (Fig. 1C; Video S1) (18). While the mechanism of syncytia formation is likely conserved from other cell types—viral proteins on the cell surface driving membrane fusion—little is understood about the way in which retraction of cellular projections occurs. Our assessment of cytoskeletal elements indicated that neurite loss is likely not due to altered gene expression, as neurofilaments, actin, and tubulin protein levels are not differentially expressed at 7 or 14 dpi (Fig. 2H through L) despite reductions in mRNA (Fig. 2B through L). In contrast, we detected significant decreases in mRNA and protein expressions of MAP2 and Tau (Fig. 3). Together, these findings suggest that the infection-induced observed reorganization of the neuronal cytoskeleton more likely involves altered microtubule-associated proteins than expression levels of microtubule subunits.

Utilizing both Pac and Col, we sought to examine how pharmacological modulation of microtubule stability would impact HCMV-induced cytoskeletal rearrangement. Both compounds are clinically approved pharmaceuticals and have been proven efficacious for the stabilization (Pac) and destabilization (Col) of microtubules in both *in vivo* and *in vitro* systems (62–65). When deciding our concentrations for each drug, we wanted to ensure an appropriate balance between the compound’s intended effects and its potential to induce cytotoxicity. The upper limits for Pac and Col were 20 nM and 10 nM, respectively, due to documented cell toxicity above that range (45, 48). We found similar results for Pac (10-20nM) and Col (10 nM), resulting in increased neuronal varicosities (Fig. 5A and D). As we intended to incubate neurons with each for a minimum of 6 days, we decided upon concentrations below those utilized in short-term studies (Fig. 5G). However, it remains possible that the modest effects we describe are due to these low dosages. For this reason, additional follow-up studies are necessary to better titer each drug for peak effectiveness. However, Pac treatment did induce some maintenance of neurite structure in treated cultures relative to the untreated infection group (Fig. 6A,B, E and F). Quantification of neurite length in Pac-treated cells demonstrated a trend toward increased neurite length relative to infected neurons treated with DMSO (Fig. 6E). In longer neurites—specifically those ≥75 µm—these changes were significant and indicate Pac’s potential for preserving neuron structure during infection (Fig. 6F). This is particularly apparent when considering the presence of longer neurites from iPSC line 1, which are not present in non-Pac-treated data sets. Together, these data suggest that there may be a potential benefit of Pac treatment in the context of HCMV neuronal infection.

In addition to the structural damage induced by infection, neurons undergo severe functional disruptions, including reductions in calcium dynamics and elimination of action potential generation (18, 55, 56). Additional research is needed to determine the impact of microtubule-modulating treatments on calcium dynamics and electrophysiological activity in HCMV-infected neurons to fully understand the potential benefits or consequences of microtubule stability. However, considering the importance of neurite structure in neuronal signaling (66, 67), we would hypothesize that even modest maintenance of structure could improve overall synaptic activity. In support of this, others have found that low-dose Pac positively impacts neuronal function in models of Alzheimer’s Disease (68), tauopathy (69), and spinal cord injury (70).

To our surprise, there was no significant disruption in virus production. Neither live imaging for eGFP expression (Fig. 7A) nor expression of the viral proteins IE1 and pp28 (Fig. 7D through F) showed a substantial reduction with Pac treatment. It is possible that with higher Pac concentration or a longer duration treatment paradigm, there would be a more obvious decrease in viral titers. However, this may come at the expense of overall neuron health. Additionally, the culture system does not contain brain-resident macrophage cells (microglia), which could alter the effects of neuronal HCMV infection (71, 72) and/or the effect of Pac and Col treatment (73, 74). As such, additional research is needed to fully understand the role that the immune system plays in HCMV-induced neuronal damage. Nevertheless, even a modest improvement in neurite outgrowth could have subsequent downstream beneficial effects on neuron health, survival, and function. Together, these data show microtubule stability as a potential modifier of HCMV neuronal pathology in neurons and may be a novel target of therapeutic intervention.

## MATERIALS AND METHODS

### Cell culture and differentiation

iPSC lines were derived from either reprogrammed skin fibroblasts or patient blood cells (Line 1: Coriell GM03814 [Fibroblast line reprogramed to iPSCs]; Line 2: WiCell PENN022i-89-1 [purchased as iPSCs; derived from blood cells]; Line 3: Coriell AG25370D [purchased as iPSCs; derived from fibroblasts]; Line 4: Coriell AG27607D [purchased as iPSCs; derived from fibroblasts]; Line 5: HB53, kind gift of Dr. Ivor Benjamin [received as iPSCs; derived from blood cells]). The utilized cell lines were chosen due to our extensive use of each line, availability, consistency of differentiation, and varying patient backgrounds. Lines 1, 3, and 4 were derived from white females at ages of 30, 81, and 69, respectively. Line 2 was obtained from a 28-year-old African American male. Line 5 was derived from a 25-year-old Caucasian male (75). Neither we nor previous studies have evaluated HCMV infection status (latency) in these individuals prior to utilizing the above cell lines. Stem cell cultures were expanded under feeder-free culture conditions using Geltrex (Gibco) basement membrane matrix. iPSCs were plated onto six-well plates and maintained with complete, daily replacements of EssentialEight medium (Thermo Fisher Scientific). Post-thaw, all iPSC cultures were passaged a minimum of three times before differentiation. All cultures were subject to quarterly mycoplasma testing to ensure freedom from contamination.

Patterning of iPSC colonies toward an NPC fate was completed utilizing dual-SMAD inhibition, as described by Chambers et al. (76). For this step, STEMdiff SMADi Neural Induction Kit (STEMCELL Technologies) and lab-prepared neural progenitor medium (Neurobasal, 10% [vol/vol] Knockout Serum Replacement [Gibco], 1% [vol/vol] 100× Non-essential Amino Acids [Gibco], 2% [vol/vol] 50× B27 Supplement [Gibco], 1% [vol/vol] 100× N2 Supplement [Gibco], 1% [vol/vol] 100× Antibiotic-Antimycotic [Gibco], 100 ng/mL Laminin [Sigma], 40 µM SB431542 [Biogems], and 0.2 µM LDN193189 [Biogems]) were used interchangeably. NPCs were cultured for a minimum of three passages in SMAD-inhibition medium (with daily changes) prior to differentiation to ensure complete and consistent generation of neural progenitors. For each passage, NPCs were dissociated using accutase (STEMCELL Technologies) to generate a single-cell suspension. Cells were then replated at a density of 2 × 10^5^ cells/cm^2^ onto Geltrex-coated six-well plates. NPCs were passaged once every 6–7 days. Between 18 and 21 days of differentiation, NPCs were dissociated and plated at a density of 1.25 × 10^5^ cells/cm^2^ in preparation for patterning toward immature forebrain neurons.

Using the STEMdiff Forebrain Neuron Differentiation Kit (STEMCELL Technologies), NPCs were directed toward a forebrain neuron cell fate. Cells were maintained under daily media changes for 6–7 days prior to final accutase dissociation and plating onto either Poly-L-Lysine- and Laminin-glassware or Geltrex-coated plasticware at varying densities (six-well plate: 5.2 × 10^4^ cells/cm^2^, 24-well plate: 1.32 × 10^5^ cells/cm^2^, and coverslips: 3.1 × 10^5^ cells/cm^2^). Upon plating, immature forebrain neurons were cultured in forebrain neuron maturation medium (BrainPhys Neuronal Medium [STEMCELL Technologies], 2% [vol/vol] 50× B27 Supplement [Gibco], 1% [vol/vol] 100× N2 Supplement [Gibco], 1%[vol/vol] 100× Antibiotic-Antimycotic [Gibco], 100 ng/mL Laminin [Sigma Aldrich], 10 ng/mL Brain-Derived Neurotrophic Factor [PeproTech], 10 ng/mL Glial Cell Line-Derived Neurotrophic Factor [PeproTech], and 1 µg/mL dibutyryl-cAMP [STEMCELL Technologies]) until use.

### Viruses

Engineered to express eGFP, BAC-TB40/E (TB40/E-eGFP) was generously provided by Felicia Goodrum (University of Arizona). This HCMV variant was generated via transfection of MRC-5 fibroblasts with both a BAC containing the HCMV genome and a UL82-encoding plasmid, as previously described (42, 43, 77). Utilizing the stocks of TB40/E-eGFP prepared in fibroblasts, ARPE-19 epithelial cells were infected and utilized to produce epithelial cell-derived TB40/E-eGFP. Viral titers were determined for epithelial-derived stocks using the method previously reported by our group (18, 60, 78) and are expressed in infectious units per milliliter (IU/mL). All neuronal infections were conducted at an MOI of 3. Viral inoculum (or phosphate buffered sailine (PBS) for mock-treated) was applied to the cell medium for 2 hours at 37°C, with constant agitation (rotary shaker plate). After exposure, cells were washed once with 1× Dulbecco’s PBS (dPBS, Gibco) to remove residual viral particles, and fresh medium was added.

### Microtubule modulation treatments

Colchicine (Col, Tocris) and paclitaxel (Pac, MilliporeSigma) working stocks were created by dissolving each compound in dimethyl sulfoxide (DMSO) to obtain concentrations ranging from 0.5 to 10 µM and 1 to 20 µM, respectively. Stocks were kept frozen at 4°C between administrations. At 21 days of differentiation, neurons were treated with 0.5–10 nM of Col or 1–20 nM Pac for 7 days. DMSO was used as a diluent control. Fresh compound was added with medium replacement at day 3. Cells were fixed for analysis 7 days post-treatment. Beginning at 51 days of differentiation, Col (2 nM), Pac (5 nM), or DMSO was added to the culture medium at a dilution of 1:1,000. When media change was required (every 3–4 days), fresh Col, Pac, or DMSO was added to the neuronal medium at 1:1,000. Cultures were treated with each compound until 58 days of differentiation, at which point they were collected for analysis.

### Immunofluorescence and live imaging

Forebrain neuron cultures were plated onto 12 mm Poly-L-Lysine (PLL, Sigma)/Laminin (Sigma)-coated coverslips at a density of 35,000 per coverslip (3.09 × 10^5^ cells/cm^2^). Cells were fixed in 4% paraformaldehyde for 15 minutes, washed once with dPBS, and stored in fresh dPBS at 4°C until use. Cell permeabilization was conducted by applying Triton X-100 solution (0.2% [vol/vol], in PBS) to each coverslip and incubating for 10 minutes at room temperature (RT). After 1× PBS wash, cells were treated with blocking buffer (5% [vol/vol] normal goat/donkey serum [NGS/NDS; dictated by secondary] in PBS) for 1 hour at RT. Primary antibody solution (primary antibodies, 5% [vol/vol] serum [NDS/NGS], 0.1% [vol/vol] Triton X-100, in PBS) was then applied to coverslips overnight at 4°C. After 4× PBS washes, cells were treated with secondary antibody solution (secondary antibodies, 5% [vol/vol] serum, 0.1% [vol/vol] Triton X-100, in PBS) and allowed to incubate for an hour at RT. The secondary antibody solution was removed, and coverslips were washed 4× with PBS to clear non-specific binding. Coverslips were mounted onto slides using Fluoromount-G Mounting Medium with DAPI (SouthernBiotech) and sealed using clear nail polish (Ted Pella Inc.). Coverslip imaging was conducted using a Zeiss LSM980 confocal microscope at various magnifications (20×–63×). Image analysis was conducted using NIS Elements (Nikon), Zen Blue (Zeiss), and ImageJ.

The following antibodies and dilutions were used for immunofluorescence during this study: chicken anti-TUJ1 (1:250–300; GeneTex), chicken anti-MAP2 (1:250–500; Invitrogen), mouse anti-MAP2 (1:250–500; Invitrogen), rabbit anti-TGN46 (1:500; Invitrogen), mouse anti-Doublecortin/DCX (1:50; Santa Cruz Biotechnology), rabbit anti-GFAP (1:1,000; Dako), goat anti-chicken IgY (H + L) AF647 (1:250, Invitrogen), goat anti-rabbit IgG (H + L) AF568 (1:500, Invitrogen), donkey anti-mouse IgG (H + L; (1:250, Invitrogen), and goat anti-chicken IgG (H + L) AF568 (1:500, Invitrogen).

Single-instance live cell imaging of virally encoded eGFP was collected using a Nikon TS100 inverted microscope. Additionally, timelapse live imaging was conducted using an IncuCyte S3 in-incubator system (Sartorius AG). Images were collected every 2 hours for 7 days, using settings to capture brightfield and green fluorescence images (ex. 440–480 nm; em. 504–544 nm). Incucyte imaging was conducted using a 20× objective, and analysis was conducted using the Incucyte 2022B revision 2 software package (video stitching, background subtraction, and eGFP quantification) and ImageJ (neurite retraction).

### Neurite and syncytia measurements

The ImageJ plugin NeuronJ was for neurite tracing and analysis. Each image consisted of a DAPI and MAP2 channel; in infected cultures, the GFP channel was referenced to determine areas of high infection. Prior to tracing, three regions of interest (ROI; 2,000 × 2,000 pixels) were overlayed onto each image. For ROIs without syncytia, five nuclei were chosen for neurite analysis. Neurites were traced from the soma to the longest branch. For ROIs containing syncytia, neurites were traced from the edge of the syncytia to the longest branch (79–81). To examine the effects of drug treatment and infection on long neurites (≥75 µm), which we identified as the median length, the total data were filtered by length using Microsoft Excel. The ImageJ freeform line tool was used to trace around the general shape of nuclei forming each syncytium. Each image consisted of a DAPI and MAP2 channel, and the GFP channel was referenced to verify the presence of syncytia. The measurement functions, shape descriptors, and area were used to obtain the measurements for circularity and area, respectively. To measure axon swellings, each image consisted of a DAPI and MAP2 channel. Prior to data collection, ROIs of 500 × 500 pixels were overlaid in areas without nuclear staining and low neurite density. Axon swellings were then counted using the ImageJ multi-point tool within each ROI.

Varicosity measures were conducted using dual-colored images labeling DAPI and MAP2. Prior to data collection, ROIs of 500 × 500 pixels were overlaid in areas without nuclear staining and low neurite density to limit intermingling of neurites. Varicosities were counted using the ImageJ multi-point tool within each ROI, and measurements were transposed into GraphPad Prism for further analysis.

To determine syncytial area and circularity, the ImageJ freeform line tool was used to trace around the general shape of nuclei forming each syncytium. As with varicosity measures, dual-channel images labeling DAPI and MAP2 were used to ascertain structure, and the GFP channel was referenced to verify that each putative syncytium was exposed to HCMV. The measurement functions “shape descriptors” and “area” were used to obtain the measurements for circularity and area, respectively, and data were analyzed within Graphpad Prism.

### Viral titers

To ensure adequate cell conditioning of all medium samples, collections occurred after a minimum of 48 hours in culture. Neuronal CM was harvested at 7 dpi for both compound- and untreated-neurons and subsequently was stored at −80°C until use. To determine the viral titers of each CM sample, stocks were serially diluted, and the resulting dilutions were applied to HCMV-naïve ARPE-19 cells. After 2 hours of exposure to undiluted inoculum, fresh medium was added to dilute the drug remaining in the conditioned medium. At 24 hours post-infection (hpi), all inoculum-containing medium was removed from the cells and replaced with fresh medium. Cells were allowed to incubate in conditioned medium for 72 hours prior to being fixed and stained for HCMV IE1 (mouse α-IE1, added 1:500 was generously provided by Tom Shenk [Princeton University]; Goat α-mouse AF488 [1:1,000]). As above, results are reported as infectious units per milliliter (IU/mL).

### Protein and DNA analyses

Reverse transcription-quantitative polymerase chain reaction (RT-qPCR) was conducted to analyze relative amounts of various elements of the neuronal cytoskeleton. Using pelleted cells from 2, 4, 7, and 14 DPI, mRNA transcripts were isolated using the standard protocol for the RNeasy extraction kit (Qiagen). Isolated RNA was treated with RQ1 RNA-free DNase (Promega) to ensure removal of contaminating genomic DNA prior to amplification. RNA transcript amplification was conducted using random hexamers and the AMV Reverse Transcriptase System (Promega). Cytoskeletal elements were assessed using the following primer sets recommended by the PrimerBank tool (82, 83): TUBB3 (5′-ATCAGCAAGGTGCGTGAGGAG-3′ and 5′-TCGTTGTCGATGCAGTAGGTC-3′), MAP2 (5′-CGAAGCGCCAATGGATTCC-3′ and 5′-TGAACTATCCTTGCAGACACCT-3′), ACTB (5′-CATGTACGTTGCTATCCAGGC-3′ and 5′-CTCCTTAATGTCACGCACGAT-3′), NEFH (5′-CCGTCATCAGGCCGACATT-3′ and 5′-GTTTTCTGTAAGCGGCTATCTCT-3′), NEFM (5′-AGGCCCTGACAGCCATTAC-3′ and 5′-CTCTTCGGCTTGGTCTGACTT-3′), NEFL (5′-ATGAGTTCCTTCAGCTACGAGC-3′ and 5′-CTGGGCATCAACGATCCAGA-3′), MAPT (5′-CCAAGTGTGGCTCATTAGGCA-3′ and 5′-CCAATCTTCGACTGGACTCTGT-3′), and GAPDH (5′-GTGGACCTGACCTGCCGTCT-3′ and 5′-GGAGGAGTGGGTGTCGCTGT-3′; Integrated DNA Technologies). Sequences were amplified using 2× SYBR Green Master Mix (Bio-Rad, Thermo Fisher). Data collection was accomplished using a Quantstudio 6 Flex real-time PCR machine (Thermo Fisher). Results for each gene were standardized against the expression of GAPDH transcripts.

Neurons intended for protein analysis were plated at a density of 5.0 × 10^6^ cells per well of a Geltrex-coated (Thermo Fisher) six-well plate (5.2 × 10^4^ cells/cm^2^). At 51 days of differentiation, cells were infected with TB40/E-eGFP and allowed to persist for another 2–7 dpi. For 14 dpi samples, cells were grown for 84 days prior to infection. Collected cells were dislodged from their basement membrane using a P1000 pipette, pelleted by centrifugation (300 × *g*; 5 minutes), and frozen at −20°C until use. Cell pellets were treated with 50–150 µL of cold, pH 7.4 lysis buffer (150 mM NaCl, 50 mM Tris-HCl [pH 8.0], 1 mM EDTA, 1% Triton X-100 [vol/vol], 1% protease inhibitor [vol/vol], and 1% phosphatase inhibitor [vol/vol]) for 20 minutes and lysed via sonication (2 × 3 s pulses; 30% amplitude). Sample concentrations were determined using the Pierce bicinchoninic acid Assay Kit (Thermo Fisher) and a Glomax Microplate Reader (Promega). Utilizing 15–30 µg of protein (consistent within blots), sample contents were resolved by SDS-PAGE on a 4%–20% acrylamide gradient gel (Bio-Rad). Separated protein bands were transferred to a polyvinylidene difluoride membrane (Millipore) using a standard wet transfer procedure and a Mini TransBlot Cell (Bio-Rad). Using Intercept (PBS) Blocking Buffer (LI-COR), membranes were blotted to reduce the possibility of non-specific binding. Next, primary antibody solution (primary antibody, Intercept Blocking Buffer, 0.2% Tween-20 [vol/vol]) was applied, and membranes were incubated overnight at 4°C with agitation. Subsequently, membranes underwent three 5-minute washes with TBS-T (tris-buffered saline, 0.1% Tween-20) prior to applying secondary antibody solution (secondary antibody, Intercept Blocking Buffer, 0.2% Tween-20 [vol/vol], and 0.02% SDS [vol/vol]) for 30 minutes at room temperature. Following several washes (3× TBS-T and 1× TBS), blots were visualized using an Odyssey CLx fluorescent imaging system (LI-COR). Primary antibodies used in this study include chicken anti-TUJ1 (1:250–500; GeneTex), chicken anti-MAP2 (1:500–1,000; Invitrogen), mouse anti-MAP2 (1:1,000, Invitrogen), mouse anti-NF68 (1:500, Sigma), rabbit anti-beta actin (1:500, Pierce), mouse anti-Tau46 (1:250–500, Cell Signaling Technologies), rabbit anti-GFP (1:1,000, Invitrogen), mouse anti-IE1 (1:1,000), and mouse anti-pp28 (1:400). Anti-HCMV antibodies were generously provided by Tom Shenk. Secondary antibodies used in this study include donkey anti-rabbit 680RD (1:2,000–3,000, LI-COR), donkey anti-mouse 800CW (1:2,000–3,000, LI-COR), donkey anti-chicken 680RD (1:3,000, LI-COR), and donkey anti-chicken 800CW (1:3,000, LI-COR).

Protein loaded into each well for western blot analysis was normalized using total protein staining. In some instances, blots were stripped and re-probed, but the total protein stain was unchanged. Reused blots are indicated as such in the appropriate figure legend. Subsequently, for blots comparing mock to HCMV at multiple timepoints (Fig. 2J through L and 3F, H, J and L), HCMV-treated samples were normalized to their mock counterpart within each timepoint. Due to this, no comparisons are made between timepoints. For blots comparing the effects of drug treatments on neurons, all protein values are normalized to the average of untreated, mock-infected values after total protein normalization.

### Statistical analysis

Data were generated from a minimum of three independent differentiations from each iPSC line, with the observer blinded to the treatment condition. All statistical analyses contained within this study were completed using the GraphPad Prism software suite. For all data sets, normality of distribution was established using the “Normality and Lognormality Tests” function, thereby allowing for appropriate selection of parametric vs nonparametric statistical tests. Should the data set fail any of the four applied assessments (D’Agostino and Pearson test, Anderson-Darling test, Shapiro-Wilk test, and Kolmogorov-Smirnov test), data were assessed in a nonparametric manner. Figure legends denote the specific statistical tests applied to each data set, with significance being defined as * <0.05 (** <0.01, *** <0.001, and **** <0.0001).

## SUPPLEMENTAL MATERIAL

Video S1 spectrum.01198-25-s0001.mp4 Syncytium formation from 1 to 7 days post-infection. Red arrowhead denotes growing syncytium over time. Green signal is HCMV-encoded eGFP.

ASM does not own the copyrights to Supplemental Material that may be linked to, or accessed through, an article. The authors have granted ASM a non-exclusive, world-wide license to publish the Supplemental Material files. Please contact the corresponding author directly for reuse.
